# Notes on Human Trials of Transcranial Direct Current Stimulation between 1960 and 1998

**DOI:** 10.3389/fnhum.2017.00071

**Published:** 2017-02-23

**Authors:** Zeinab Esmaeilpour, Pedro Schestatsky, Marom Bikson, André R. Brunoni, Ada Pellegrinelli, Fernanda X. Piovesan, Mariana M. S. A. Santos, Renata B. Menezes, Felipe Fregni

**Affiliations:** ^1^Neural Engineering Laboratory, Department of Biomedical Engineering, City College of New York, City University of New YorkNew York, NY, USA; ^2^Biomedical Engineering Department, Amirkabir University of TechnologyTehran, Iran; ^3^Neurology Service Hospital de Cínicas de Porto Alegre, Department of Internal Medicina, Universidade Federal do Rio Grande do Sul (UFRGS)Porto Alegre, Brazil; ^4^Hospital Moinhos de VentoPorto Alegre, Brazil; ^5^Laboratory of Neuroscience (LIM27), Department and Institute of Psychiatry, University of São PauloSão Paulo, Brazil; ^6^Faculdade de Ciências Médicas da Santa Casa de São Paulo, School of Medical SciencesSão Paulo, Brazil; ^7^Departamento de Medicina - Fortaleza, Universidade de Fortaleza, Centro de Ciências da SaúdeCeará, Brazil; ^8^Laboratory of Neuromodulation, Physical Medicine and Rehabilitation Department, Spaulding Rehabilitation Hospital, Harvard Medical SchoolBoston, MA, USA

**Keywords:** tDCS, electric stimulation therapy, human, brain, review

## Abstract

**Background**: Transcranial direct current stimulation (tDCS) is investigated to modulate neuronal function including cognitive neuroscience and neuropsychiatric therapies. While cases of human stimulation with rudimentary batteries date back more than 200 years, clinical trials with current controlled stimulation were published intermittently since the 1960s. The modern era of tDCS only started after 1998.

**Objectives**: To review methods and outcomes of tDCS studies from old literature (between 1960 and 1998) with intention of providing new insight for ongoing tDCS trials and development of tDCS protocols especially for the purpose of treatment.

**Methods**: Articles were identified through a search in PubMed and through the reference list from its selected articles. We included only non-invasive human studies that provided controlled direct current and were written in English, French, Spanish or Portuguese before the year of 1998, the date in which modern stimulation paradigms were implemented.

**Results**: Fifteen articles met our criteria. The majority were small-randomized controlled clinical trials that enrolled a mean of approximately 26 subjects (Phase II studies). Most of the studies (around 83%) assessed the role of tDCS in the treatment of psychiatric conditions, in which the main outcomes were measured by means of behavioral scales and clinical observation, but the diagnostic precision and the quality of outcome monitoring, including adverse events, were deficient by modern standards. Compared to modern tDCS dose, the stimulation intensities used (0.1–1 mA) were lower, however as the electrodes were typically smaller (e.g., 1.26 cm^2^), the average electrode current density (0.2 mA/cm^2^) was approximately 4× higher. The number of sessions ranged from one to 120 (median 14). Notably, the stimulation session durations of several minutes to 11 h (median 4.5 h) could markedly exceed modern tDCS protocols. Twelve studies out of 15 showed positive results. Only mild side effects were reported, with headache and skin alterations the most common.

**Conclusion**: Most of the studies identified were for psychiatric indications, especially in patients with depression and/or schizophrenia and majority indicated some positive results. Variability in outcome is noted across trials and within trials across subjects, but overall results were reported as encouraging, and consistent with modern efforts, given some responders and mild side effects. The significant difference with modern dose, low current with smaller electrode size and interestingly much longer stimulation duration may worth considering.

## Introduction

Transcranial direct current stimulation (tDCS) consists of applying a weak direct current on the scalp, a portion of which crosses the skull (Datta et al., 2009) and induces cortical changes (Fregni and Pascual-Leone, 2007; Nitsche et al., 2008). The investigation of the application of electricity over the brain dates back to at least 200 years, when Giovanni Aldini (Zaghi et al., 2010) recommended galvanism for patients with deafness, amaurosis and “insanity”, reporting good results with this technique especially when used in patients with “melancholia”. Aldini also used tDCS in patients with symptoms of personality disorders and supposedly reported complete rehabilitation following transcranial administration of electric current (Parent, 2004).

These earliest studies used rudimentary batteries and so were constant voltage, where the resulting current depends on a variable body resistance. Over the 20th century, direct voltage continued to be used but most testing involved pulsed stimulation, starting with basic devices where a mechanical circuit that intermittently connected and broke the circuit between the battery and the subject and evolving to modern current control circuits including Cranial Electrotherapy Stimulation and its variants (Guleyupoglu et al., 2013). Interest in direct current stimulation (or tDCS) resurged with the studies of Priori et al. (1998) and Nitsche and Paulus (2000) that demonstrated weak direct current could change cortical response to Transcranial Magnetic Stimulation, thereby indicating that tDCS could change cortical “excitability”. Testing for clinical and cognitive modification soon followed (Fregni et al., 2005, 2006). Developments and challenges in tDCS research, including applications in the treatment of neuro-psychiatrics disease since 1998 have been reviewed in detailed elsewhere (Brunoni et al., 2012).

This historical note aims to explore earlier data on human trial using current controlled stimulation (tDCS) before 1998 with the goal of informing ongoing understanding and development of tDCS protocols. As expected, we found variability in the quality of trial design, data collection and reporting in these earlier studies. Nonetheless, many clinical findings are broadly consistent with modern efforts, including some encouraging results but also variability across subjects. We also describe a significant difference in dose with lower current, smaller electrodes and much longer durations (up to 11 h) than used in modern tDCS.

## Methods

### Literature Search

For our searching methodology, we included articles that: (a) investigated the clinical effects of transcranial direct current stimulation; (b) were published before 1998; (c) human studies; (d) written in English, Spanish, Portuguese or French; (e) controlled current for stimulation. We also excluded articles if they were reviews or meta-analysis, as well as studies that involved invasive procedures or other methods of electrical stimulation.

To identify relevant studies, we searched PubMed using the keywords (*brain polarization*), (*transcranial direct current stimulation*) and (*electric stimulation therapy*) along with (*brain*). We also searched the reference list of all selected articles to identify other relevant articles that we might have missed during the primary PubMed search. Initially, AP and PS conducted the search but, if there were any unresolved issue, FF was consulted. Most of the articles were not available online; therefore they were retrieved at *Francis A. Countway* *Library of Medicine* (Harvard, Cambridge, MA, USA).

The data was collected using a semi-structured form for each study. The following variables were extracted: (a) title; (b) year of publication; (c) Journal; (d) number of participants in the study; (e) their pre-existing condition; (f) medications; (g) intensity of the applied current; (h) duration of each session; (i) number of sessions; (j) total duration of stimulation; (k) position of the electrodes; (l) electrode size; (m) the strategy of stimulation; (n) clinical effects; (o) side effects; (p) trial design; (q) conclusion; and (r) main outcome. Some of these data were shown in Table 1. Because we only found 15 articles fulfilling the inclusion criteria, and included articles had with incomplete and variable reporting details, it was not prudent to conduct quantitative analysis.

**Table 1:** **tDCS studies published between 1960 and 1998**.

Study	N	Disease	Design	Electrode montage	Intensity (mA)	Duration of stimulation	Electrode	Findings	Side effects
Lippold et al. (1964), UK	32	Depression/ Schizophrenia	Uncontrolled double-blind	Anodes over each eyebrows and cathode over right knee	0.1 to 3 mA*	0.5 to 5** h (duration of stimulation varied in subjects based on their condition and improvement).	1.26 cm^2^ Chloride silver discs covered with saline-soaked gauze	In scalp-positive polarization patients became more alert and more involved with the environment; in scalp-negative polarization quietness and withdrawal was seen. They have often found an effect at 0.25 mA for each anode whereas there had repeatedly been no effect at 0.15 mA scalp positive stimulation***.	Tremor during scalp-positive, nausea, sleepiness

Costain et al. (1964), UK	24	Depression	Controlled double-blind, crossover	Anodes over each eyebrows and cathode over one knee	0.25 for each anode % current was started from 0.1 for each eyebrow and gradually increased	8 h per day for 12 days	1.26 cm^2^ Silver discs covered with saline-soaked gauze	Improvement of anxiety, agitation and somatic symptoms.	Faint, blue flashes, skin sensitivity, mild headaches

Redfearn et al. (1964),UK	29	Refractory depression	Open label	Anodes over each eyebrows and cathode over one knee	0.1 to 0.25 for each anode	0.5 to 11** (duration for each person was based on side effects), 5 times a week for 6 months.	1.26 cm^2^ Chlorided silver discs covered with saline-soaked gauze	13 cases showed clinical improvement that lasted only 1–2 days. It has been suggested that a dosage of 0.4 mA in each lead for period on 8 h per day was more effective in many patients.	Mild headache, skin sensitivity

Ramsay et al. (1966), USA	20	Depression	Open label	Anodes over each eyebrows and cathode over one knee	0.15 to 0.3 for each anode	4 to 6** h per day. Total stimulation time varies.	–	14 definitely improved, 4 equivocal improved, 2 did not improve.	Few side effects reported (does not mention which)

Herjanic et al. (1967)	20	Depression/ Schizophrenia	Uncontrolled open label	-	0.1 to 0.5	–	–	All patients improved their depressive symptoms.	None reported

Lifshitz and Harper (1968), USA	5	Schizophrenia	Controlled double-blind crossover	Anodes over eyebrows and cathodes over homolateral thighs.	0.33 for each channel of stimulation	6 h per day for two weeks only on week days followed by two week rest period.	Pure silver electrodes covered by surgical gauze soaked with normal saline. Anode = 1 × 2.5 cm and cathode = 2 × 4 cm	No significant effects either for scalp positive or scalp negative stimulation.	Skin irritation was fairly marked for 3 patients. Skin lesion consisted of erythema, papules and pustules which principally appeared under negative electrode.
Sheffield et al. (1968), Australia	6	Healthy	Controlled double blind	Anodes over eyebrows and cathode over one leg	0.25 for each lead % current started from 0.03 mA and gradually increased in 90 minutes	3 h, each person was stimulated twice (positive and negative) in different days.	Chlorided silver discs covered with saline soaked lint pads. Anode= 0.5 inch diameter, cathode= 0.75 inch diameter.	Happier and more alert with scalp-positive polarization but results don’t show significant changes in mood in subjects compared to control.	Moody and sleepy with scalp-negative polarization

Carney et al. (1970), Australia	119	Depression	Open label, uncontrolled	–	0.25	–	–	Improvement in excited behavior and mood; relapse on stopping treatment and improvement on recommencing.	None reported

Arfai et al. (1970), USA	19	Depression	Controlled double blind clinical trial	Anodes over eyebrows and cathodes over thighs	0.25 for each independent channel	8 h/day during 6 days each week (totally 12 applications)	Chlorided silver discs	No significant effects.	None reported

Hall et al. (1970), USA	18	Healthy	Controlled double-blind	Anodes over each eyebrows and cathode over knee	0.15 and 0.3 for each lead	2 h, each person was stimulated 3 times (scalp positive, scalp negative and sham) in different days.	Metallic mesh electrodes. Skin was rubbed by alcohol and local anesthetic was used.	No significant effect.	None reported

Baker et al. (1970), Rhodesia	107	Depression	Random group of patients treated with brain polarization.	Anodes over each eyebrows and cathode over upper arm or forearm	0.4 for each lead % current started with 0.2 mA and gradually reached 0.4 in half an hour	5 h** per day for 6 to 8 sessions.	Silver plates covered with lint soaked in saline and gel was used for skin Anode= 10 cm^2^ and cathode= 20 cm^2^.	84% reported sustained improvement. Anxiety was not relieved.	Skin sensitivity, tachycardia and migraine

Nias and Shapiro (1974), UK	1	Schizophrenia with depression	Double blind controlled clinical trial	Anodes over each eyebrows and two cathodes attached to right knee	0.4 for each lead	3–4 h per day for 14–120 sessions.	–	Improvement with negative and worsening with positive stimulation	Tingling on the forehead.
	1	Alcoholism with depression			0.5 for each lead			Improvement with positive stimulation
Elbert et al. (1981a), Germany	48	Healthy	Single-blinded	Anode over vertex and cathodes over earlobes	0.26	1 h in a session (half of task was done in cathodal and the rest was done in anodal stimulation).	1.5 cm diameter Silver discs	Vertex positive current tends to develop faster reaction times and higher skin conductance responses than vertex-negative currents.	None reported
Elbert et al. (1981b), Germany	32	Healthy	Single-blinded	Anode over vertex and cathode over collarbone to both sides which were linked	0.25	1 h in a session (half of it was anodal and the other half was cathodal stimulation).	1.5 cm diameter Silver discs	Results suggest that subjects reacted after a shorter interval when negative pole was applied compared to positive stimulation.	None reported

Korsakov (1989), Russia	48	Schizophrenia	Open label clinical trial	Anode over occipital cortex OR anode over frontal CORTEX cathode = mastoid	0.05 to 0.2	–	Silver cup electrodes	Cathodal on occipital cortex increased visual sensitivity (discrimination of the brightness of a pair of light flashes), anodal decreased.	None reported

*= 3 mA was used just for one person and it was applied while putting local anesthetic under electrode. **= Device was portable and patients could go about their normal hospital business and returning to the lab at pre-arranged times. ***= They had two other failed trials before the present study. The essential difference between this trial and two others were electrodes placed over eyebrows, currents were lower and they were passed for much longer time.

### Terminology

For the purpose of this study we combine typical terminology used in modern tDCS with literature with conventions in classic literature. tDCS always requires a positive (anode) and negative (cathode) electrode on the body. The term “active” indicates the electrode which is considered by the investigator to exert behavioral effects, presumably by modulating cortex under the electrodes, while “return” electrode indicate the counter polarity electrode which is presumed to have no or less consequential effect. The anode electrode is presumed to generate an excitatory influence, while the cathode a local inhibitory influence. This concept pervades historical to modern tDCS design, though modern neurophysiology, imaging and computational modeling suggest that how and which brain regions are modulated by tDCS is much more complex. One electrode must always be on the head. In modern literature, an electrode below the head is “extra-cephalic” and typically placed on the forearm. In older literature, “scalp-positive” or “scalp-negative” is used to indicate the use of an extra-cephalic electrode, typically placed on the hand or foot with the anode or cathode, respectively, on the head. For example, “scalp-positive” is comparable to “active anode electrode with extra-cephalic return”. For all the limitations in this terminology, here we respect nomenclature as used in the original reports. Electrode dimensions are assumed to refer to contact area between the electrolyte (sponge) and skin.

## Results

**Figure 1:**
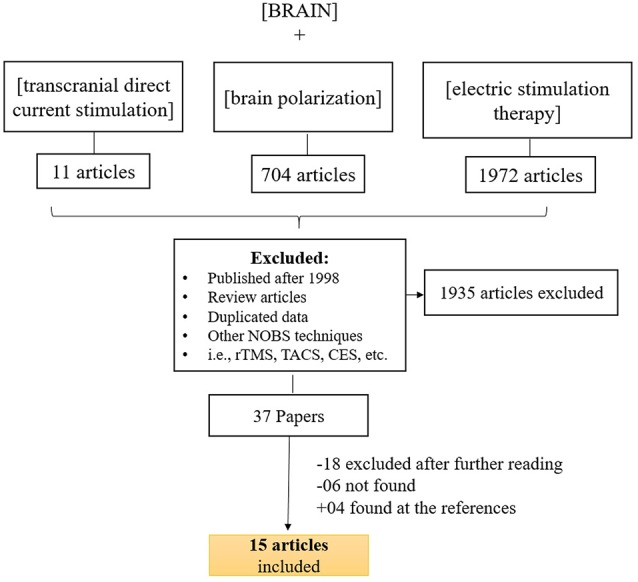
**Search strategy for inclusion, exclusion criteria of this study.** To identify relevant studies, we searched PubMed using the keywords (*brain stimulation*), (*transcranial direct current stimulation*) ad (*electric stimulation therapy*) along with (*brain*).

**Figure 2:**
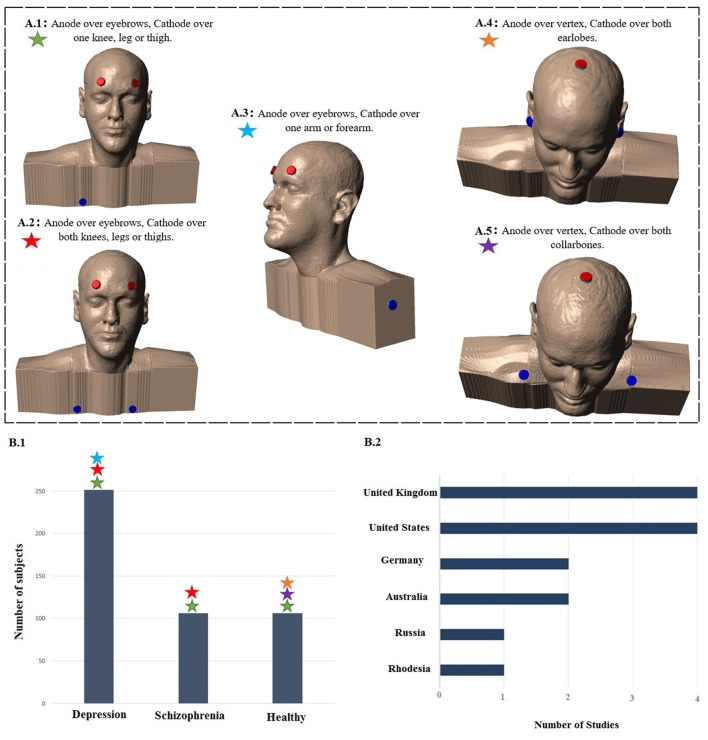
**Summary of study parameters on human trials using transcranial direct current stimulation (tDCS) in old literature (from 1960 to 1998).** Models of commonly used montages of tDCS in early studies **(A)**; red: anode electrode(s), blue: cathode electrode(s). Total number of subjects in each group of patients participating in studies using aforementioned montages **(B.1)** and leading countries conducting tDCS studies in early stage with number of published articles **(B.2)**.

Figure 1 displays the diagram of search strategy and its results. Table 1 indicates the final selected studies. Given these 15 articles, the oldest where current was controlled was written in 1964. The majority of articles were small studies with number of patients varying from 1 to 107 (mean, 26 subjects). Approximately half of the studies (8 out of 15) were randomized controlled trials, but there were also two single blind and five open the studies. Most of the studies involved patients with psychiatric disorders, mainly major depression and schizophrenia (Figure 2B). Only four studies were performed using exclusively healthy subjects. Eight out of 15 studies were performed in United Kingdom and United States (Figure 2B). Positive results were obtained in most of the analyzed studies (Table 1).

### tDCS Parameters

The intensity of electric current varied between studies. The median of most commonly used intensity was 0.33 mA for each anode; typically ranging from 0.1 mA (Redfearn et al., 1964) to 0.5 mA (Nias and Shapiro, 1974) for each anode. However, Lippold and Redfearn (1964) applied 3 mA in one single patient. The most common electrode montage was: active electrode(s) above eyebrow and reference electrode in extra-cephalic position (e.g., leg, hand; Figure 2A). The active electrodes were most commonly placed in the frontal—especially supraorbital—but also in occipital areas of the scalp and vertex. Apart from the leg and arm, other locations for the return electrode were also used such as the mastoid bone or collarbone. Historically, the approach of applying a stimulation over orbital fissures originated from two other failed trials conducted by Lippold and Redfearn (1964) and trial and error in electrode placement, current intensity and stimulation duration. They found that largest modification in mood and alertness would be produced when anode is placed over an orbital fissure and cathode at an extra-cephalic location (e.g., leg, thigh or arm). The essential differences between the two failed trials and the successful one was location of electrodes, lower applied current with longer duration of stimulation (Lippold and Redfearn, 1964) which was used in most of studies on depression afterwards.

Only 8 out of 15 studies specified the precise dimensions of the electrodes; in those ones the smallest active electrode was of 0.1 cm^2^ and the smallest reference electrode was of 0.2 cm^2^. The reference electrode area was often larger than the active ones, from approximately 30% (Lifshitz and Harper, 1968) to 50% bigger (Baker, 1970), but in some cases was the same (Elbert et al., 1981a). The use of a larger return electrode compared to active electrode is in line with modern conventions (Woods et al., 2016), though even the larger active and return electrodes are smaller than used in modern tDCS.

Most of studies employed several sessions of stimulation, with a median of 14 sessions. The quantity of session varied from one single to 120 sessions. The median of the total duration of stimulation was 30 h. Redfearn et al. (1964) conducted the longest study, with 960 h as the total time of stimulation.

The mean duration of session was 4.5 h (4 h and 30 min) with a maximum of 11 h (Redfearn et al., 1964) of electrical stimulation. Due to the long duration of stimulation in several studies, the devices were portable and patients were able to move around the hospital or go home (Lippold and Redfearn, 1964; Redfearn et al., 1964; Ramsay and Schlagenhauf, 1966; Baker, 1970). The regimen of sessions varied across articles—daily sessions or several days interval between sessions. In average, stimulation protocols consisted of applying 0.33 mA for 6 h per session that was continued up to 14 days.

In most included studies, stimulation apparatus was made of low voltage dry batteries in a pack with a potentiometer manually adjusted to produce a constant current. In a later study (Elbert et al., 1981b), an optocoupled system driven by the analog output provided constant current which had a ramp up period of 6 s to increase current from 0 mA to 0.25 mA. In all the studies, electrodes were metallic, either pure silver or silver chloride disks covered with saline soaked gauze or lint. Electrode contact and current was checked in pre-arranged times especially in studies with longer duration of stimulation.

### Clinical and Side Effects

Twelve studies reported positive results. With the exception of Arfai et al. (1970), all other studies with melancholic or depressive patients showed some positive results using tDCS. The most common side effects reported were headache and skin sensitivity. Half of the studies did not mention any side effects.

## Discussion

Across the limited historical use of tDCS between 1960 and 1998, there was little standardization of electrical parameters of stimuli. The lack of methodological rigidity on some parameters such as reference electrode position, number of sessions, the target area, current strength, electrode size and duration of each session might explain some contradictory findings between the studies. There was often limited information on subject inclusion and recruitment, in one case, not even the place of origin of the study was apparent (Herjanic and Moss-Herjanic, 1967).

The values of the current intensity used in the selected historical tDCS trials, from 0.1 mA to 0.5 mA (median 0.33 mA) for each anode(s), were overall lower than those ones used contemporarily in clinical trials, which vary between 1–3 mA (median 2 mA; Bikson et al., 2016). Potentially maximum current was constrained by hardware limitations (battery voltage), especially with the need for portability (small size and weight) and long duration operation (hours per session). Smaller electrodes were used in historical tDCS trials, but this may have marginal or no effects in resulting brain current density, compared to the linear loss with reduced current intensity (Miranda et al., 2009). Nitsche et al. (2008) demonstrated that, when stimulations durations are limited to several minutes, an intensity of 0.6 mA is required to induce a significant change in average cortical excitability detectable by TMS. Total stimulation charge was determined by the current and time. The neurophysiological consequence of lower-intensity stimulation but with longer period (e.g., hours) is unknown. In most cases included here the total charge applied (e.g., 4.5 h times 0.25 mA for each anode = 8100 mC^1^) was above that is used in modern tDCS (e.g., 2 mA for 20 min = 2400 mC). The side effect profile of the included historical trials, to the extent they were monitored and reported was mild.

Most of the studies placed the active electrode above the eyebrow and the reference one on the leg, or on the arm. This position of the active electrode approximates locations used in modern human trials. However, the “reference” electrode is now more commonly placed on the head; extra-cephalic “return” electrodes are sometimes used. Modern computational modeling studies suggest the use of extra-cephalic electrode produce significant current flow in deep and mid-brain structures (DaSilva et al., 2011). Indeed, Redfearn et al. (1964) suggested that highest current density in extra-cephalic stimulation could be in brainstem and supported it by evidence of respiratory depression caused by applying 3 mA cathodal stimulation in a normal subject.

Historical tDCS trials employed from 1 to 120 sessions with a median of 14 sessions, and a median of 4.5 h (20 min to 11 h) of electrical stimulation per session, resulting in a total duration of the trial with a median of 30 h (150 min to 960 h). Currently, it is known that stimulation duration of 20–30 min is more than enough to induce cortical excitability chances and consequently clinical improvements rather than hours of stimulation that would compromise patient’s compliance in clinical daily practice (imagine a patient using tDCS for hours at home).

### The Use of Outcomes

The Hamilton Depression Rating Scale—HDRS (Hamilton, 1960), recognized as the gold standard in modern depression trials, although contemporary to the majority of early tDCS reviewed was not adopted. Rather early tDCS studies favored clinical outcomes and depression self-rating scales, more subjective and of difficult comparability. Only one study used the HDRS (Arfai et al., 1970). Other more objective measures used in depression trials were: laboratory changes (norepinephrine, serotonin, beta-endorphin and cholinesterase) and cardiac frequency. In the other conditions addressed, also subjective and objective outcomes assessment was conducted. Among the validated outcomes, the Benton Visual Retention Test (Benton, 1946) was used to evaluate the improvement in short-term memory in alcoholic patients. Tests of reaction to light stimuli were performed within a schizophrenic group of patients. Other studies took into account laboratory changes in hormone levels, self-report scales and several clinical outcomes such as remission of symptoms, improvement in terms of re-hospitalization and/or further treatment and medical evaluation.

### Trial Design

The majority of the retrieved articles consisted of double blind controlled clinical trials, which is considered as the “gold standard” for intervention studies. On the other hand, some of them were inadequately reported, therefore making difficult to assess their quality. In a few of these studies, the blinding status was not clearly defined especially in those allocating patients with major depression. In fact, without an appropriate blinding, the results might be biased by a decrease of the placebo effect, as well as an increase of the number of false-positive results and over-estimate of the magnitude of an association. Another aspect to take into account is the high electrical density used that might have precluded blinding process. In most historical trials, the number of subjects was relativity small (indicating cautious interpretation of the results), this remains the case in modern tDCS pilot trials on new indications.

### Adverse Effects

It is difficult to draw a reliable evaluation of the side effects from these works as the majority of articles did not post how many healthy subjects or patients were affected, and when multiple intensities were used did not correlate adverse events with intensity. There were no reports of subjects needing to terminate a session or receive medical care for injury. In contemporary tDCS trials, the most common side effects using standard protocols and montages—all transitory—are a mild tingling followed by itching and headache (Brunoni et al., 2011). Autonomic reactions are considered unlikely according to recent systematic review (Schestatsky et al., 2013). Historical studies lacked systematic questionnaire searching for adverse events, which might underestimate detection of occurrence.

### Synopsis

In conclusion, we found 15 studies with semi-systematic approaches before the year of 1998, considered the time point of contemporary tDCS. For dosage, the use of multi-hour stimulation session, albeit with modestly reduced current intensity is a significant deviation from modern protocols. The use of supra-orbital active electrode(s) with an extra-cephalic return is another feature in these older studies, though rarely used in modern tDCS.

It is difficult to draw firm meta-conclusions from the analysis of the 15-included studies. This is due to lack of information regarding patient’s diagnosis and stimulation parameters as well as varied scientific rigor in design study. The most common type of patients addressed was from the psychiatric field. The occurrence of unusual adverse events i.e., papules, pustules and faint, might be related to longer duration of stimuli and higher density but also other conditions apart from the stimulation itself, such as stimulus-induced anxiety and unrelated events in patients.

## Author Contributions

All authors contributed equally to this work.

## Conflict of Interest Statement

MB has equity in Soterix Medical Inc. The City University of New York has patents on Brain Stimulation with MB as inventor. The other authors declare that the research was conducted in the absence of any commercial or financial relationships that could be construed as a potential conflict of interest.

## Footnotes

^1^Mili-Coulomb (mC).
